# Sterile versus non‐sterile gloves during cystoscopy: A randomized prospective single‐blind study

**DOI:** 10.1002/bco2.284

**Published:** 2023-09-03

**Authors:** Mika Fukushima, Masaaki Imamura, Masanori Ito, Kei Muraoka, Michiko Fukasawa, Masatoshi Kumagai, Ryo Yabusaki, Masakatsu Ueda, Yusuke Shiraishi, Tetsuya Noguchi, Koji Yoshimura

**Affiliations:** ^1^ Department of Urology Shizuoka General Hospital Shizuoka Japan; ^2^ Shizuoka City Shizuoka Hospital Shizuoka Japan

**Keywords:** non‐sterilization, bladder cancer, cystoscopy, sterilization, urinary tract infection

## Abstract

**Objective:**

The objective of this study is to evaluate the need for sterile gloves during cystoscopy by comparing the incidence of UTI symptoms between patients in whom the procedure is performed with non‐sterile gloves with those performed with non‐sterile gloves.

**Patients and Methods:**

This study had a randomized, prospective, single‐blind design and included patients aged >20 years who underwent cystoscopy in either of two outpatient clinics between September 2015 and November 2021. The patients were allocated to a sterile group or a non‐sterile group. Only the urologists were aware of whether or not the gloves were sterile. The patients were instructed to report any symptoms suggestive of UTI after cystoscopy.

**Results:**

A total of 1258 patients were enrolled in the sterile group and 1376 in the non‐sterile group. Symptoms of UTI were reported by six patients (0.48%) in the sterile group and six (0.44%) in the non‐sterile group. The between‐group difference was not statistically significant (p = 0.88).

**Conclusion:**

It is not necessary to use sterile gloves during routine cystoscopy.

## 1 INTRODUCTION

Flexible cystoscopy is used to evaluate haematuria, lower urinary tract symptoms and bladder cancer and is one of the most common urological procedures performed in urology clinics.^1^ Urinary tract infection (UTI) is one of the complications associated with cystoscopy but occurs in fewer than 5% of patients regardless of whether or not antibiotics are administered.^2^, ^3^ Therefore, the current American Urological Association (AUA) guideline for antibiotic prophylaxis in patients undergoing urological procedures does not recommend the use of antibiotics before cystoscopy.^4^

Other measures that are important in prevention of infection when using medical devices such as a cystoscope are sterilization of the device and avoiding transmission of microorganisms from the urologist to the patient.^5^ Although a sterilization method has been established for cystoscopes,^6^ the best method for avoiding transmission of microorganisms is unclear. Clinicians routinely wear sterile gloves to prevent infection during cystoscopy.^6^ However, there is no clear evidence of the superiority of sterile gloves over non‐sterile gloves in terms of the risk of UTI after cystoscopy. Indeed, for minor surgical procedures in fields other than urology, such as incisions for treatment of skin cancer, non‐sterile gloves have been reported to be safe as sterile gloves.^7^ Therefore, it may be safe to wear non‐sterile gloves during cystoscopy. In this study, we evaluated the incidence of UTI symptoms after cystoscopy according to whether sterile or non‐sterile gloves are worn by the urologist during the procedure.

## 2 PATIENTS AND METHODS

The study was approved by the institutional review boards of Shizuoka General Hospital and Shizuoka City Shizuoka Hospital in Japan. Written informed consent was obtained from all study participants. All procedures were performed in accordance with the ethical standards laid down by our institutional research committee.

The study had a randomized, prospective, single‐blind design and enrolled patients aged >20 years who underwent cystoscopy in an outpatient clinic at either of the participating institutions between September 2015 and November 2021. Using a computer‐generated randomization list, the patients were allocated in a ratio of 1:1 to undergo cystoscopy performed by a urologist wearing sterile gloves or non‐sterile gloves. Only the urologists knew the sterilization status of the gloves.

The exclusion criteria were as follows: inability to understand the reason for the examination or lacking self‐judgement ability; a history of symptomatic UTI after cystoscopy performed using sterile gloves; need for urethral anaesthesia (i.e., intraurethral injection of xylocaine jelly) during cystoscopy; high risk of UTI (e.g., uncontrolled diabetes, on immunosuppressant therapy or presence of an indwelling foreign body, e.g., a pacemaker); other infectious disease; cystoscopy performed for a reason other than intravesical observation, such as biopsy and/or cauterization and inappropriate for participation in the study in the opinion of the attending urologist. Antibiotic usage immediately after cystoscopy was not allowed. Therefore, if patients received antibiotic therapy within several days of cystoscopy, their data were excluded from the analysis.

Cystoscopy was performed after disinfecting the genital area with benzalkonium chloride. All patients underwent cystoscopy using a 16.5‐Fr flexible cystoscope (CYF‐5A®; Olympus Medical Systems, Tokyo, Japan) and had routine urological indications for cystoscopy, such as follow‐up of non‐invasive bladder cancer after transurethral resection. After each cystoscopy procedure, the cystoscope was sterilized by manual scrubbing, an ultrasonic wash using OER‐3® (Olympus) followed by exposure to disinfectant solution (Acecide®; Olympus) and rinsing.

The patients were instructed to report any symptoms suggestive of a bacterial infection in the lower urinary tract if they occurred within 2–14 days after cystoscopy. Symptoms of interest included urinary frequency, difficult urination and fever of 38°C or higher with left or right back pain and/or testicular pain. The primary endpoint was the incidence of UTI symptoms after cystoscopy.

### 2.1 Statistical analysis

We assumed that UTI symptoms would occur in 4% of participants in the sterile group and 6% of those in the non‐sterile group. A sample size of 1863 participants in each group was estimated to be required for a power of 80% at an alpha value of 0.05. Allowing for an approximately 5% dropout rate, we planned to recruit 2000 patients for each group. The between‐group difference in the primary endpoint was examined using the chi‐squared test. All statistical analyses were performed using JMP version 10.0 (SAS Institute Inc., Cary, NC, USA).

## 3 RESULTS

**FIGURE 1:**
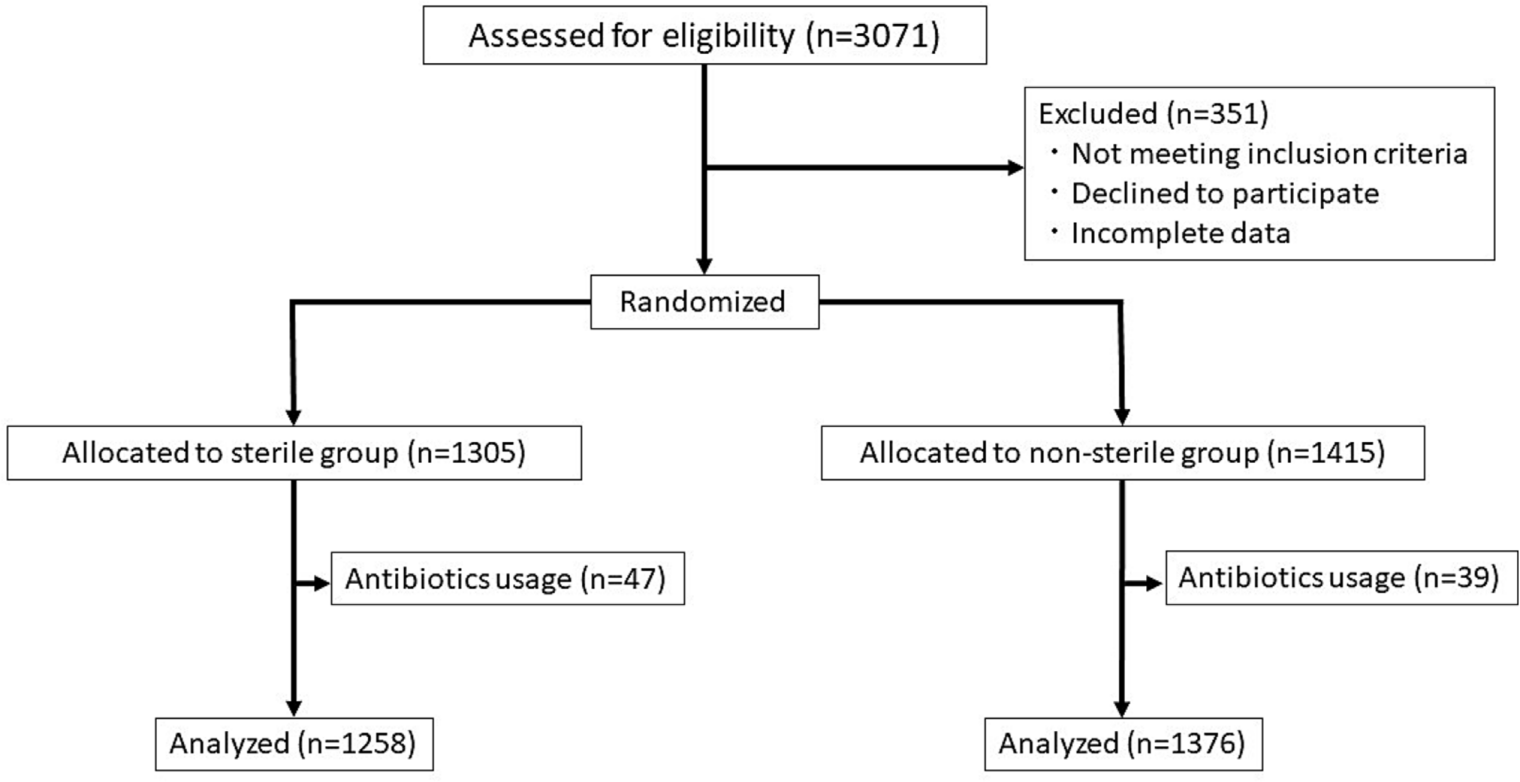
Consolidated Standards of Reporting Trials (CONSORT) diagram showing the flow of patients through the study.

Although we initially planned to recruit a total of 4000 patients, patient accrual was terminated in November 2021, partly because of repeated extensions for recruitment purposes and partly because of the recruitment difficulties encountered during the COVID‐19 (coronavirus disease 2019) pandemic. As shown in the Consolidated Standards of Reporting Trials (CONSORT) flow diagram (Figure 1), 3071 patients were finally enrolled (sterile group, *n* = 1258; non‐sterile group, *n* = 1376). There were no statistically significant differences in baseline characteristics between the two groups (Table 1).

**TABLE 1:** Baseline characteristics in both study groups.

	Overall	Sterilized group	Non‐sterilized group	*P*‐value
	(*n* = 2634)	(*n* = 1258)	(*n* = 1376)	
Age (years)				
Median	72 (28–92)	72 (28–92)	72 (28–92)	0.3
Sex				
Female	459	213	246	0.52
Male	2175	1045	1130	
Medical disease				
Diabetes mellitus (%)	455 (17.3)	224 (17.8)	231 (16.8)	0.49
Cardiovascular disease (%)	613 (23.3)	301 (23.9)	312 (22.7)	0.45
Neurological disease (%)	166 (6.3)	87 (6.9)	79 (5.7)	0.22
Urinary findings				
Bloody urine (%)	315 (12.0)	157 (12.5)	158 (11.5)	0.43
Purulent urine (%)	387 (14.7)	180 (14.3)	207 (15.0)	0.59
Observation of cystoscope				
Tumour in the bladder	272 (10.3)	136 (10.8)	136 (9.9)	0.34

Six patients (0.48%) in the sterile group and six (0.44%) in the non‐sterile group reported symptoms suggestive of UTI. The between‐group difference was not statistically significant (*p* = 0.88; Table 2). The most common symptom was difficulty on urination, which was observed in 11 cases. Urinalysis and/or urine culture in these cases confirmed bacterial infection in three patients in the sterile group and three in the non‐sterile group; all six patients had fever. Eleven of the 12 patients with symptoms of UTI were treated with antibiotics. Symptoms in the remaining patient resolved without treatment (Table 3).

**TABLE 2:** Incidence of UTI symptoms in both study groups.

	Sterilized group	Non‐sterilized group	Odds ratio (95% CI)	*P*‐value
	(*n* = 1258)	(*n* = 1376)		
Symptoms of suggestive UTI				
Yes (%)	6 (0.48)	6 (0.44)	0.91 (0.29–3.02)	0.88
No (%)	1252 (99.5)	1370 (99.6)		

Abbreviation: UTI, urinary tract infection.

**TABLE 3:** UTI symptoms in the sterile and non‐sterile group.

Case	Age	Sex	Time to onset (day)	Symptoms	Bacteria	Treatment
UTI symptoms in the sterile group						
1	68	M	4	Fever, difficulty on urination	Unknown	Follow‐up
2	59	M	3	Difficulty on urination	Negative	Antibiotics
3	74	F	2	Urinary frequency, difficulty on urination	*Escherichia coli*, *Streptococcus agalactiae*	Antibiotics
4	73	M	6	Fever, difficulty on urination	*Enterococcus faecalis*	Antibiotics
5	75	F	3	Urinary frequency, difficulty on urination	*Escherichia coli*, *S. agalactiae*	Antibiotics
6	72	M	4	Fever, testicular pain	Negative	Antibiotics
UTI symptoms in the non‐sterile group						
1	81	F	4	Difficulty on urination	Unknown	Antibiotics
2	73	M	14	Urinary frequency, difficulty on urination	Unknown	Antibiotics
3	71	M	13	Difficulty on urination	Unknown	Antibiotics
4	79	M	2	Fever, difficulty on urination	Citrobacter species	Antibiotics
5	76	M	4	Fever, difficulty on urination	*Enterococcus faecalis*	Antibiotics
6	77	M	2	Fever, difficulty on urination	*Klebsiella aerogenes*	Antibiotics

Abbreviation: UTI, urinary tract infection.

## 4 DISCUSSION

In this study, the incidence of UTI symptoms was much lower than expected in both groups. To some extent, this finding could be attributed to our study protocol; whereby, symptoms of UTI were self‐reported. However, the rate of febrile UTI was also similar in the two groups, suggesting that the incidence of UTI was low in both groups.

To the best of our knowledge, this study is the first to identify a low incidence of UTI after cystoscopy performed with non‐sterile gloves. There have been several reports on the association between non‐sterile urethral catheter insertion procedures and infection, including a review, which found that a non‐sterile procedure did not affect the infection rate in the 14 days after insertion of an indwelling catheter.^8^

Studies performed in fields other than urology, for example in minor skin surgery, have found a low incidence of surgical site infection after use of non‐sterile gloves and that non‐sterile gloves seem to protect against infection as effectively as sterile gloves.^9^, ^10^ Moreover, studies of uncomplicated wound repair in the emergency room have found a low infection rate regardless of whether sterile or non‐sterile gloves are used.^11^, ^12^ Interestingly, one previous study found that the incidence of surgical site infection with non‐sterile gloves was lower in patients who underwent procedures involving simple skin excision than in those who underwent reconstructive surgery.^13^ These data suggest that non‐sterile gloves can prevent transmission of infection during minimally invasive procedures. Considering that cystoscopy is a minimally invasive urological procedure, sterile gloves may not be necessary. Our results are compatible with this suggestion. Furthermore, given that the aforementioned review found that tap water was sufficient for cleaning the genitalia at the time of insertion of an indwelling catheter,^8^ even disinfection with benzalkonium chloride might be unnecessary for cystoscopy.

The use of non‐sterile gloves has the advantages of being cost‐effective and time‐saving.^12^ In terms of cost‐effectiveness, a previous study reported that the cost of non‐sterile gloves was one‐tenth that of sterile gloves and that the total cost associated with cystoscopy using non‐sterile gloves were less than half than that using sterile gloves.^14^ Simple methods that use fewer items are important in terms of time‐saving. Sterile techniques require time‐consuming collection of several materials before the procedure^12^ and could be avoided by using non‐sterile methods. In addition to the low infection rate, these two advantages support the use of non‐sterile gloves when performing cystoscopy.

This study has several limitations. First, the target patient accrual could not be met in a timely manner, to some extent because of the COVID‐19 pandemic. However, we were able to recruit almost three‐quarters of the planned number of patients. Second, UTI was diagnosed based on self‐reporting of subjective symptoms rather than objective data. Almost all patients who reported symptoms were subsequently diagnosed with UTI by urine culture; however, some had negative urine culture results because of early treatment after onset of symptoms. Using this method for the diagnosis of UTI might have affected the accuracy of our data. However, only four patients with symptoms of UTI had a negative urine culture and would not have affected our conclusion regarding the low incidence of UTI in this study. Third, exclusion of patients at high risk for UTI might have contributed to our low incidence of UTI. Nevertheless, our findings suggest that the incidence of UTI is low when non‐sterile gloves are used during cystoscopy in low‐risk patients. Further studies are needed in patients at high risk for UTI. Finally, the sterilization status of the gloves used in this study was known to the clinicians, which might have led to them performing cystoscopy more carefully when using non‐sterile gloves. However, gloves are commercially available materials, and the likelihood of being able to perform a double‐blind study using sterile and non‐sterile gloves with the same appearance would be slim.

Despite these limitations, this study has some strengths in the office urology setting in that it had a randomized prospective design and included patients undergoing cystoscopy who were blinded to the sterilization status of the gloves worn by their urologist. Our main finding that the incidence of UTI is low whether the gloves used during cystoscopy are sterile or non‐sterile indicates that non‐sterile gloves are safe to use during cystoscopy.

## 5 CONCLUSION

This prospective study found that the use of non‐sterile gloves was non‐inferior to the use of sterile gloves in terms of the rate of post‐cystoscopy UTI. Using non‐sterile gloves could be an option for routine cystoscopy.

## AUTHOR CONTRIBUTIONS

*Data analysis*: Mika Fukushima. *Manuscript writing*: Mika Fukushima. *Data collection*: Masaaki Imamura, Masanori Ito, Kei Muraoka, Michiko Fukasawa, Masatoshi Kumagai, Ryo Yasbusaki, Masakatsu Ueda, Yusuke Shiraishi, Tetsuya Noguchi and Koji Yoshimura. *Manuscript revisions*: Masaaki Imamura and Koji Yoshimura. *Study conception and design*: Koji Yoshimura.

## CONFLICT OF INTEREST STATEMENT

None.

## References

[1] Trail M , Cullen J , Fulton F , et al. Evaluating the safety of performing flexible cystoscopy when urinalysis suggests presence of “infection”: results of a prospective clinical study in 2350 patients. Eur Urol Open Sci. 2021;31:28–36. 10.1016/j.euros.2021.06.014 34467238 PMC8385291

[2] García‐Perdomo HA , López H , Carbonell J , Castillo D , Cataño JG , Serón P . Efficacy of antibiotic prophylaxis in patients undergoing cystoscopy: a randomized clinical trial. World J Urol. 2013;31(6):1433–1439. 10.1007/s00345-013-1034-2 23412704

[3] Herr HW . The risk of urinary tract infection after flexible cystoscopy in patients with bladder tumor who did not receive prophylactic antibiotics. J Urol. 2015;193(2):548–551. 10.1016/j.juro.2014.07.015 25046618

[4] Lightner DJ , Wymer K , Sanchez J , Kavoussi L . Best practice statement on urologic procedures and antimicrobial prophylaxis. J Urol. 2020;203(2):351–356. 10.1097/JU.0000000000000509 31441676

[5] Rutala WA , Weber DJ . Disinfection, sterilization, and antisepsis: an overview. Am J Infect Control. 2016;47:A3–A9. 10.1016/j.ajic.2019.01.018 31146848

[6] Safiullah S , Lama DJ , Patel R , Clayman RV . Procedural module: flexible cystoscopy. J Endourol. 2018;32(S1):S2–S6. 10.1089/end.2017.0706 29774810

[7] Michener M , Xia Y , Larrymore D , McGraw T , McCarthy S . A comparison of infection rates during skin cancer excisions using nonsterile vs sterile gloves: a prospective randomized pilot study. J Cosmet Dermatol. 2019;18(5):1475–1478. 10.1111/jocd.12860 30661299

[8] Lockwood C , Page T , Conroy‐Hiller T , Florence Z . Management of short‐term indwelling urethral catheters to prevent urinary tract infections. JBI Libr Syst Rev. 2004;2:1–36. 10.11124/01938924-200402080-00001 27820018

[9] Heal C , Sriharan S , Buttner PG , Kimber D . Comparing non‐sterile to sterile gloves for minor surgery: a prospective randomized controlled non‐inferiority trial. Med J Aust. 2015;202(1):27–31. 10.5694/mja14.00314 25588441

[10] Brewer JD , Gonzalez AB , Baum CL , Arpey CJ , Roenigk RK , Otley CC , et al. Comparison of sterile vs nonsterile gloves in cutaneous surgery and common outpatient dental procedures. JAMA Dermatol. 2016;152(9):1008–1014. 10.1001/jamadermatol.2016.1965 27487033

[11] Perelman VS , Francis GJ , Rutledge T . Sterile versus nonsterile gloves for repair of uncomplicated lacerations in the emergency department: a randomized controlled trial. Ann Emerg Med. 2004;43(3):362–370. 10.1016/j.annemergmed.2003.09.008 14985664

[12] Zwaans JJM , Raven W , Rosendaal AV , Van Lieshout EMM , Van Woerden G , Patka P , et al. Non‐inferiority of non‐sterile gloves and dressing versus sterile gloves, dressings and drapes for suturing of traumatic wounds in the emergency department: a multicentre randomised controlled trial. Emerg Med J. 2022;39(9):648–649. 10.1136/emermed-2021-211540 35882525

[13] Rogues AM , Lasheras A , Amici JM , Guillot P , Beylot C , Taïeb A , et al. Infection control practices and infectious complications in dermatological surgery. J Hosp Infect. 2007;65(3):258–263. 10.1016/j.jhin.2006.09.030 17244515

[14] Carapeti EA , Andrews SM , Bentley PG . Randomised study of sterile versus non‐sterile urethral catheterisation. Ann R Coll Surg Engl. 1994;76:59–60. PMC2502653 8659977

